# The Sensory Quality and Volatile Profile of Dark Chocolate Enriched with Encapsulated Probiotic *Lactobacillus plantarum* Bacteria

**DOI:** 10.3390/s18082570

**Published:** 2018-08-06

**Authors:** Milica Mirković, Sanja Seratlić, Kieran Kilcawley, David Mannion, Nemanja Mirković, Zorica Radulović

**Affiliations:** 1 Faculty of Agriculture, University of Belgrade, Nemanjina 6, Belgrade 11080, Serbia; zradulovic@agrif.bg.ac.rs; 2 Teagasc Food Research Centre, Moorepark, Fermoy, Co. Cork P61, Ireland; sanja.seratlic@teagasc.ie (S.S.); kieran.kilcawley@teagasc.ie (K.K.); david.mannion@teagasc.ie (D.M.); 3 Institute for Molecular Genetics and Genetic Engineering, University of Belgrade, Vojvode Stepe 444, Belgrade 11000, Serbia; nemanjamirkovic@imgge.bg.ac.rs

**Keywords:** chocolate, probiotics, encapsulation, viability, sensory analysis, volatiles

## Abstract

Cocoa and dark chocolate have a wide variety of powerful antioxidants and other nutrients that can positively affect human health. Probiotic dark chocolate has the potential to be a new product in the growing number of functional foods. In this study, encapsulated potential probiotic *Lactobacillus plantarum* 564 and commercial probiotic *Lactobacillus plantarum* 299v were added in the production of dark chocolate. The results show very good survival of probiotic bacteria after production and during storage, reaching 10^8^cfu/g in the first 60 days and over 10^6^cfu/g up to 180 days. No statistically significant difference (*p* > 0.05) in chemical composition and no major differences in the volatile profiles between control and experimental chocolate samples were observed, indicating no impact of probiotic bacteria on compositional and sensory characteristics of dark chocolate. The sensory evaluation of control and both probiotic dark chocolate samples showed excellent sensory quality after 60 and 180 days of storage, demonstrating that probiotics did not affect aroma, texture and appearance of chocolate. Due to a high viability of bacterial cells and acceptable sensory properties, it can be concluded that encapsulated probiotics *Lb. plantarum* 564 and *Lb. plantarum* 299v could be successfully used in the production of probiotic dark chocolate.

## 1. Introduction

Specialized chocolate shops and supermarkets have a wide range of different chocolates but probiotic chocolate remains a novel product. Probiotic bacteria are usually delivered within dairy products, such as fermented milks and cheeses, where bacteria perform a major role in the development of the final product characteristics. However, lactose intolerance, allergenic milk proteins [1,2] and high fat content are the major drawbacks related to the intake of dairy products, especially for health-conscious consumers. Additionally, required refrigeration and relatively short shelf-life of dairy products represent also limitations in their use. In this context, the evolution of a new probiotic product could be of significant importance. Cocoa and chocolate have been suggested as a good food medium for functional healthy ingredients, because they are rich sources of catechins (flavan-3-ols, or flavanols), epicatechin and procyanidins, which are polyphenols that have the ability to act as antioxidants [3,4,5], showing cardio protective [6,7,8] and antidepressant effects [9]. Moreover, Possemiers et al. [10] revealed that chocolate was a better probiotic carrier than dairy products for intestinal delivery, because bacterial survival rates through gastro-intestinal tract conditions were four times higher in chocolate than in dairy products.

Dark chocolate is made from cocoa (in form of paste, powder or butter) and sugar (added as an emulsifier and sometimes as a flavour) and it does not contain milk, while milk chocolate contains whole-milk powder, including emulsifying agents and very often some flavourings [11,12]. Therefore, dark chocolate with 75% cacao was chosen as a probiotic carrier in this study. The use of a dense food matrix which contains a higher content of protective ingredients has additional advantages over typical dairy probiotic products.

Chocolate can be defined as a product made primarily of cocoa solids and cocoa fats, although since 1999 up to 5% of its content can be comprised of vegetable fat alternatives, such as palm oil, shea, sal, illipe or mango kernel butter [11]. Different chocolate flavours can be obtained by varying the temperature and time when roasting the beans or adjusting the quantities of cocoa solids and/or adding non-chocolate ingredients. The specific flavour of dark chocolate is primarily due to a very rich volatile fraction which is composed of a mixture of hundreds of volatile and non-volatile compounds. There are many descriptive studies that have identified more than 600 volatile compounds in chocolate products and cocoa, mainly pyrazines, amines and amides, acids, esters and hydrocarbons [4,13,14]. Composition of dark chocolate depends on the genotype of the cocoa bean and the processes used during chocolate production: fermentation, drying, roasting and conching [14]. Cocoa bean fermentation is an essential step in the development of key volatile fractions (alcohols, esters and fatty acids) and the formation of flavour precursors (amino acids and reducing sugars). Molecules generated during cocoa bean fermentation, such as pyrazines, aldehydes (cocoa, nut aroma), esters (fruity aroma) and polyphenols (astringent and bitter taste) are mainly responsible for cocoa and chocolate flavour formation [15,16]. Important flavour-active compounds produced during fermentation include 2-methylbutanoate, tetramethyl-pyrazine and other pyrazines. Bitter notes are evoked by caffeine and threobromine, while other flavour precursor components derived from amino acids during fermentation include phenyl-acetaldehyde, 3-methylbutanol, 2-methyl-3-(methyldithio)furan, 2-ethyl-3,5-dimethyl-methylpyrazine and 2,3-diethyl-5-methylpyrazine [17]. Flavour development continues during drying (moisture decrease in cocoa bean from around 60% to around 7.5%), where the characteristic brown colour is developed and the levels of acidity and astringency are reduced by decreasing the volatile acids and total polyphenol content. The next crucial step for flavour improvement is cocoa roasting and it involves Maillard reactions (chemical reactions between amino acids and reducing sugars under heat treatment), which provide the desirable flavour and colour formation. Roasting also affects the concentration of polyphenols and their ability to interact with proteins, which causes a decrease in astringency [18,19]. Conching (agitating chocolate at >50 °C) is a final stage in flavour development during chocolate manufacture and is important in determining the final flavour characteristics and texture formation [4,20].

Within the general quality of chocolate, sensory quality is the most important parameter and it is therefore necessary that the sensory attributes should not be altered by the addition of probiotic bacteria.

The aim of this study was to examine the viability of two encapsulated spray dried bacteria *Lactobacillus plantarum* 564 (potential probiotic) and *Lactobacillus plantarum* 299v (commercial probiotic) in dark chocolate after production and during 360 days of storage at room temperature and their influence on volatile compounds and sensory characteristics of final product. To increase process efficiency and improve survival of bacterial cells, an encapsulation process was applied using spray drying. It is a low-cost process that achieves dried powder of small particle size with optimal moisture content and fast production of large quantities of viable cells [21]. It is envisaged that this research will provide a knowledge platform for the commercial development of probiotic dark chocolate.

## 2. Materials and Methods

### 2.1. Strains and Culture Conditions

A potential probiotic strain *Lb. plantarum* 564, isolated from artisanal Serbian white brined cheese [22] and a commercial probiotic strain *Lb. plantarum* 299v (DSM, Heerlen, The Netherlands) were used in this study. Strain *Lb. plantarum* 564 belong to the strain collection of the Department for Industrial Microbiology, Faculty of Agriculture, University of Belgrade, Serbia. This strain was selected according to technological properties and probiotic potential which is tested by Radulović et al. [22]. Both strains were cultured in MRS broth (Merck, Darmstadt, Germany) at 37 °C in anaerobic conditions.

### 2.2. Encapsulation Process of Lactobacillus plantarum

The spray drying encapsulation process was performed according to the method described by Radulović et al. [23]. Overnight cultures (300 mL) were centrifuged (4500× *g*, 15 min, 15 °C), the pellet was washed twice in 50 mM K_2_HPO_4_ (pH 6.5), re-suspended in 300 mL of sterilised reconstituted skim milk (20% *w*/*v*) and spray-dried with a laboratory scale spray-dryer (Büchi mini spray dryer model B-290, Flawil, Switzerland) using the constant inlet air temperature of 170 °C and the outlet temperature of 80 °C.

### 2.3. Enumeration of Encapsulated Lactobacillus plantarum

Enumeration of spray dried probiotic cells was performed after reconstitution of 1 g of spray dried powder in 9 mL of 2% (*w*/*v*) sodium citrate and appropriate decimal dilution was plated on MRS agar (Merck, Darmstadt, Germany). The plates were incubated at 37 °C for 48 h in anaerobic conditions (Gas Pak, Merck, Darmstadt, Germany).

### 2.4. Chocolate Production

Production of dark chocolate was carried out in the semi-industrial scale in the factory of confectionery products Soko Štark, Belgrade, Serbia. A control sample without and two experimental samples containing spray dried probiotic strains (*Lb. plantarum* 564 and *Lb. plantarum* 299v) were produced in three replicate trials according to Laličić-Petronijević et al. [24]. Powders of spray dried probiotic bacteria were added to the chocolate masses after tempering when temperature was lower than 40 °C, by blender mixing for 5 min. Aiming to provide required number of probiotic cells in chocolate samples (10^8^cfu/g), 10 g of powder of spray dried cells were added per kg of dark chocolate, after which chocolate masses were moulded, cooled, removed from the form and packed in aluminium foil and paper blanks and stored at 20 °C for 360 days.

### 2.5. Viability of Probiotic Bacteria

Viable cell counts of both probiotic bacteria were determined by the standard plate method and values expressed as colony forming units per gram (cfu/g) of chocolate. Ten grams of dark chocolate was homogenized in 90 mL saline solution (0.9% NaCl) in a Stomacher apparatus (Lab Blender Stomacher 400, Seward, West Sussex, UK). Serial dilutions were prepared and appropriate dilutions were plated on MRS agar (Merck, Darmstadt, Germany). Total bacterial counts were determined after 48 h of incubation at 37 °C under anaerobic conditions (Gas Pak, Merck, Darmstadt, Germany). Viability of both bacterial strains were analysed in triplicate immediately after production and after 60, 90, 180, 270 and 360 days of storage at 20 °C.

### 2.6. Chemical Analysis of Chocolate

Chemical composition of dark chocolate samples (moisture, protein, fat, ash and carbohydrate content) was determined according to the AOAC methods [25].

### 2.7. Characterisation of Volatile Flavour Compounds by Head Space Gas Chromatography Mass Spectrometry

The volatile profile of the chocolate samples was analysed by static head space solid phase microextraction (HS-SPME) gas chromatography mass spectrometry (GCMS).

Each sample (5 g) was weighed into a 20-mL headspace glass amber vial with a screw top and a silicone/PTFE septum (Apex Scientific, Maynooth, Kildare, Ireland). Sample introduction was accomplished using a CTC Analytics CombiPal autosampler. Samples were equilibrated at 40 °C in a controlled temperature agitator for 10 min at 500 rpm (5 s on/off) prior to exposure of the SPME 50/30 µm Carboxen^TM^/divinylbenzene/polydimethylsiloxane (CAR/DVB/PDMS) fibre. The fibre was fully exposed to the headspace of the sample at a depth of 1 cm at 40 °C at 350 rpm for 20 min. The fibre was desorbed for 2 min at 250 °C in split-less mode onto a Varian 450 gas chromatograph with an 1177 injector (Aquilant Scientific Ltd., Dublin, Ireland) using a merlin microseal and a SPME liner. The column was a DB-5ms (60 m × 0.25 mm × 0.25 µm) (Agilent Technologies, Cork, Ireland). The extracted compounds were cyrofocussed using liquid carbon dioxide at −60 °C directly onto the column. The temperature of the column oven was initially held at −60 °C for 2 min, increased to 20 °C at 20 °C/min, held for 20 min, then increased to 110 °C at 10 °C/min, followed by an increase to 290 °C at 15 °C/min, yielding at total run time of 47.0 min. The carrier gas was helium, held at a constant flow of 1 mL/min. The detector was a Varian 320 triple quad mass spectrometer (Aquilant Scientific Ltd., Ireland), used in single quadrupole mode. Compounds were identified using mass spectra comparisons to the NIST 2008 mass spectral library and from an internal data base of standards created over time. An auto-tune of the GCMS was carried out prior to the analysis to ensure optimal GCMS performance. A set of standards was also run at the start and end of the sample set to ensure the MS was performing within specification. Each sample was analysed in triplicate after 180 days of storage.

### 2.8. Sensory Analysis

The sensory quality of dark chocolate samples with probiotic bacteria during storage was analysed using a modified scoring method for sensory evaluation of chocolate as described by Popov-Raljić and Laličić-Petronijević [26]. Sensory evaluations included the following properties: appearance (form, colour, gloss and surface), mechanical properties (structure, break, hardiness and chewiness), geometrical properties (sandiness), surface properties (moisture and lubricity), aroma (odour, taste) and other dynamic properties (solubility), as shown in Table 1. For each sensory property, the Weight Coefficient was determined based on the influence of each attribute on overall sensory quality. A scoring range from 1.00 to 5.00, with the possibility of assigning half and quarter points was applied. Quality category was determined in scores spans; samples which were evaluated with less than 2.5 points were considered as unsatisfactory, 2.5–3.5 good, 3.5–4.5 very good and 4.5–5 excellent quality. Sensory evaluation of all three chocolate types was performed by seven experienced assessors familiar with the product. All panellists met the criteria specified by the ISO standards for selection, training and monitoring of assessors [27,28].

The sensory analysis of the probiotic chocolate samples was performed after 60 and 180 days of storage at room temperature of 20 °C.

### 2.9. Statistical Analysis

Statistical significance was tested by means of ANOVA analysis and the differences between individual mean values were tested using the Fisher’s least significant difference (LSD) test. Significant differences were considered for *p* < 0.05. Calculations were made with STATISTICA 6.0 PL software for Windows (StatSoft Inc, Tulsa, OK, USA).

The principal component analysis of sensory and volatile data was analysed using the Unscrambler Software, version 9.7 (CAMO ASA, Trondheim, Norway).

## 3. Results and Discussion

### 3.1. Cell Count of Spray Dried Probiotic Bacteria

Bacterial count of spray dried cells of potential probiotic *Lb. plantarum* 564 was 10.20 log cfu mL^−1^, while number of spray dried cells of *Lb. plantarum* 299v was 10.36 log cfu mL^−1^. Obtained results indicate that optimal conditions were achieved during spray drying process. Similar results were obtained in our previous studies [23,29].

### 3.2. Viability of Probiotic Bacteria

According to the definition given by FAO/WHO [30] probiotics are “live microorganisms which, when administrated in adequate amounts, confer a health benefits on the host”. To have a positive effect in the intestinal tract, probiotics should be able to survive low pH, gastric acid and bile secretions and should succeed in competing with the resident intestinal microbiota. The bacteria should also be able to selectively stimulate the growth and/or activity of bacteria in the colon [31,32]. Probiotic bacteria have been reported to have numerous health benefits such as balancing of intestinal microbiota, stimulation of immune system, blood cholesterol reduction, enhancement of the digestibility of protein and vitamin synthesis, anti-bacterial activities, treatment of lactose intolerance, food-related allergies, amongst others [33,34,35,36]. Most of probiotic bacteria belong to the *Lactobacillus* genera and *Bifidobacterium* strains [37]. However, variety of food grade lactic acid bacteria, have been evaluated for their potential probiotic ability. Furthermore, some of autochthonous facultative heterofermentative lactobacilli isolated from traditional dairy products, such as *Lactobacillus casei*, *Lactobacillus plantarum*, *Lactobacillus rhamnosus* can also have probiotic properties [38].

Many destabilizing factors in food production could affect viability of probiotic bacteria. In production of dark chocolate, the factors such as temperature, oxygen, osmotic pressure, water activity and so forth could be critical for bacterial cell survival. Therefore, it is very important to incorporate probiotic bacteria in dark chocolate mass at very low temperature during manufacturing process, so their viability will not be impaired. In this experiment, probiotic bacteria were added after the process of tempering when the temperature reached around 30 °C. However, chocolate composition, such as protein and fat content, sugar concentration, including high buffering capacity, remains an important factor affecting the viability of probiotic bacteria [39], not only after production but also during storage.

Based on the development of functional foods such as probiotic dark chocolate, one of the main goals is achieving the desired amount of probiotic bacteria in dark chocolate during the production and storage in order to provide a therapeutic effect on human health. To have a beneficial effect on health, probiotics must be viable in the product at the time of consumption. The threshold cell number should be above 10^6^cfu/g during manufacturing process and storage [40,41]. The viability of probiotic bacteria can be affected by many factors, particularly physicochemical properties of dark chocolate (fat and protein content, type of sugars, etc.) and addition of certain food ingredients (stabilizers, sweeteners, etc.) [42]. Different approaches that increase the viability of probiotic bacteria have been adopted, including appropriate selection of acid- and bile-resistant strains, use of oxygen impermeable containers, two-step fermentation, incorporation of micronutrients and microencapsulation [43]. Microencapsulation by spray-drying is presumably the most effective and economic method. Also, spray drying is suitable for large-scale, industrial applications [44]. Although high temperature of hot air can be a disadvantage of spray-drying, proper readjustments of processing conditions can achieve better viability of probiotic bacteria [45,46]. According to the literature, an air outlet temperature of 80 to 85 °C was optimal for spray drying to preserve *Lb. paracasei* and *Lb. salivarius* strains with probiotic potential [47,48]. Our previous studies [29] demonstrated a high viability and survival rate of spray dried *Lb. plantarum* 564 cells while using a constant inlet air temperature of 170 °C and outlet temperature of 80 °C, indicating successful use of applied spray drying conditions as a method of bacterial preservation in the present research.

**Figure 1:**
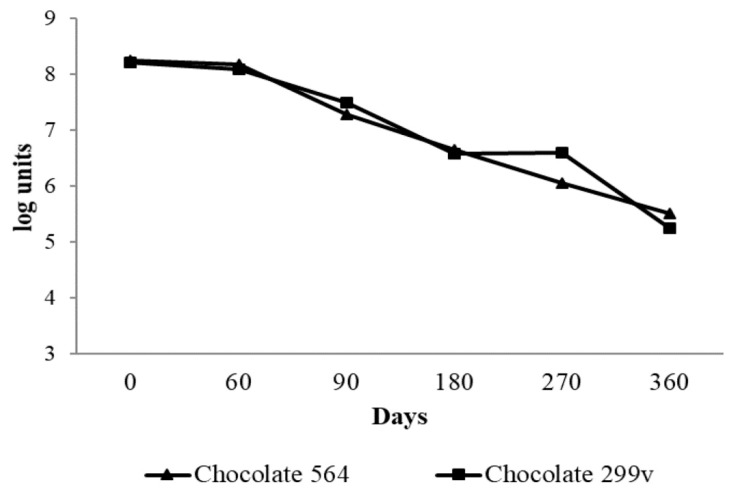
Viability of spray dried *Lb. planatrum* 564 and commercial probiotic *Lb. plantarum* 299v in dark chocolate during storage.

According to the results, the spray dried *Lb. plantarum* 564 and *Lb. plantarum* 299v cells remained viable for up to 6 months, having counts of over 10^6^cfu/g (Figure 1). The present study demonstrated very good survival of both probiotic bacteria in dark chocolate after production and during storage at room temperature, especially in first 90 days. During the first two months, the cell numbers of both potential and commercial spray dried probiotic bacteria were above 8 log units. After 90 days of storage, there was a slight decrease in viability of both *Lb. planatrum* 564 and 299v (7.28 and 7.49 log cfug^−1^, respectively). Afterwards, the cell numbers of probiotic bacteria gradually decreased and after 360 days the numbers of probiotic bacteria in both experimental chocolates were marginally above 5 log units.

Nebesny et al. [49] reported that lyophilized lactic acid bacteria *Lactobacillus casei* and *Lactobacillus paracasei* had very good survival rate in dark chocolate during storage at both 4 °C and 18 °C, where the total number of live cells maintained at functional level of 7 log units even after 12 months of storage. Laličić-Petronijević et al. [24] also observed that the number of lyophilized cells of probiotic bacteria *Lactobacillus acidophilus* NCFM^®^ remained at the level of 7 log units per gram of dark chocolate during 180 days of storage at 20 °C. Obtained results are also in accordance with findings of Mandal et al. [39], who reported above 8 log cfu/g of *lactobacilli* in probiotic chocolate during 60 days of storage, while Aragon-Alegro et al. [50] detected over 7 log cfu/g of *Lb. paracasei* in probiotic chocolate mousse stored 28 days under refrigerated conditions. In the present study, 6.5 log units of both strains were present in chocolate after 6 months and the cell number of commercial *Lb. plantarum* 299v strain remained at the same level after 9 months of storage. The decrease of bacterial cell number below the threshold of 6 log cfu/g in further storage period indicates that chocolate samples with *Lb. planatrum* 564 and commercial probiotic 299v cannot be called “probiotic chocolate” after 180 and 270 days of storage, respectively.

### 3.3. Chemical Composition of Dark Chocolate

Table 2 shows the chemical composition of three dark chocolate variants. Presented data are the mean values of three replicate trials. Results of statistical analysis showed that there was no statistically significant difference (*p* > 0.05) in moisture, protein and fat content between control and probiotic chocolate samples. Also, the slight difference in ash content between control and probiotic sample with *Lb. plantarum* 299v was not statistically different (*p* > 0.05), nor was the slight difference in carbohydrate content between control and probiotic chocolate with *Lb. plantarum* 564.

According to the results, it can be concluded that there was no statistically significant difference (*p* > 0.05) in chemical composition between tested chocolate samples, which means that spray dried probiotic bacteria did not make an impact on the main chemical composition of dark chocolate. These important findings are in accordance with other literature data [24,51].

### 3.4. Volatile Analysis

Volatile profile of control and probiotic dark chocolate is presented in Table 3.

In total, 36 volatile compounds were identified, consisting of mainly aldehydes (7), alcohols (6), ketones (6), pyrazines (5), with lower amounts of lactones (3), acids (3), esters (2), terpenes (2), phenol (1) and furan (1). Apart from two compounds (2-heptanol and isoamyl acetate), all the remaining volatiles were present in all three chocolate variants, indicating that there were no major differences in the volatile profiles between the samples, nor did the majority of volatile compounds differ significantly (*p* > 0.05) between the experimental variants and the control. The major difference detected between the samples was that the experimental samples (chocolate 564 and 229v) did not contain 2-heptanol and the control sample did not contain isoamyl-acetate.

**Figure 2:**
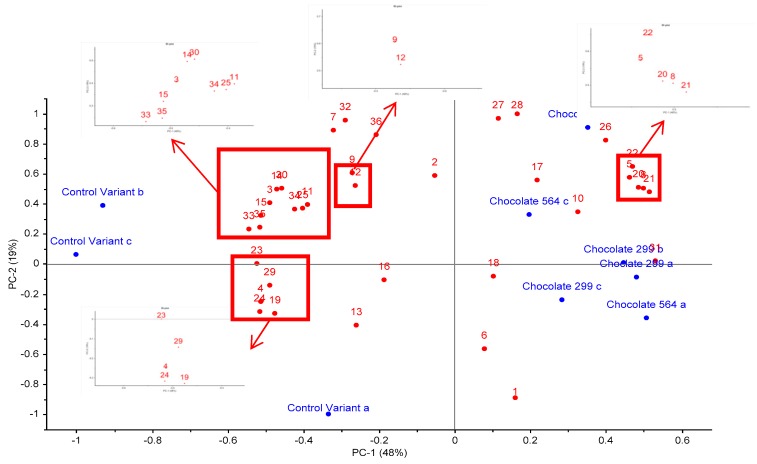
Principal component analysis (PCA) bi-plot of volatile compounds detected in dark chocolate with and without probiotic bacteria. Compounds are: 1. ethanol, 2. acetone, 3. isobutanal, 4. butanone, 5. 3-methyl-butanal, 6. acetic acid, 7. acetoin, 8. hexanal, 9. 2,3-butanediol, 10. isoamyl acetate, 11. 1,6-heptadien-4-ol, 12. isovaleric acid, 13. 2-heptanone, 14. 2-methyl-butanoic acid, 15. 2-heptanol, 16. butyrolactone, 17. benzaldehyde, 18. beta-pinene, 19. phenol, 20. 2-pentylfuran, 21. 2-ethyl-6-methylpyrazine, 22. trimethylpyrazine, 23. 2-ethyl-1-hexanol, 24. limonene, 25. pantolactone compound, 26. acetophenone, 27. 2-ethyl-3,6-dimethylpyrazine, 28. tetramethylpyrazine, 29. 2-nonanone, 30. nonanal, 31. trans-5-methyl-2-isopropyl-2-hexen-1-al, 32. phenylethylalcohol, 33. 2-methyl-3,5-diethylpyrazine, 34. phenethyl acetate, 35. d-octenolactone, 36. ethylvanillin.

Figure 2 is a principal component analysis (PCA) bi-plot of the variation in volatile profile between each sample in triplicate. The overall variance is 67%, of which 48% relates to PC1 and 19% to PC2. It is apparent that the control sample replicates (A, B & C) are all on the negative side of PC1, while the experimental probiotic samples (A, B & C replicates of chocolate 564 and 229v) are on the positive side of PC1. Some variation between the three replicates is evident but that is to be expected with HS-SPME using non-homogenous samples.

Overall, the experimental samples (chocolate 564 and 229v) were differentiated from the control by containing higher abundances of isodihydrolavandulal, 2-penyl furan, hexanal, 2-ethyl-6-methyl pyrazine, acetophenone, tetramethylpyrazine and 3-methyl-butanal. On the other hand, control samples contained higher abundance of butanone, 2-nonanone, 2-ethyl-1-hexanol, d-octenolactone and 2-methyl-3,5-diethylpyrazine.

### 3.5. Sensory Analysis

Dark or plain chocolate contains no less than 35% cocoa but nowadays there is a trend for the chocolate production with higher percentage of cocoa (such as 55%, 70%, 85% and >85%). The more cocoa the product contains, the less sugar it has and therefore the stronger intensity of cocoa flavour and the more bitter the chocolate [12]. In this study, dark chocolate samples were produced with 75% cocoa.

The panellists have evaluated the appearance of all three chocolate variants (control and experimental probiotic chocolates) by using a pair of small tongs for lifting the sample and the appearance observation. Then by taking a square of each sample and after keeping it for 20 s in a mouth, the taste was analysed by identifying the gustatory, olfactory and physical sensations. While gustatory sensations can register the bitterness, acidity and sugary content of a chocolate, the olfactory sensations are more sensitive and can register the full aromatic palette of cacao, such as grilled, roasted, spicy or smooth notes. Physical characteristics include chocolate texture, melting quality, graininess or smoothness, as well as the whole mouth feel [12].

After production and during the storage of probiotic dark chocolates, the metabolic activity of probiotic bacteria could influence some textural properties, appearance, taste and aroma. Moreover, the powder addition of spray dried bacteria could have an impact on texture with their granulation, appearance and texture. Also, powder of spray dried bacteria could affect the colour of final product.

**Figure 3:**
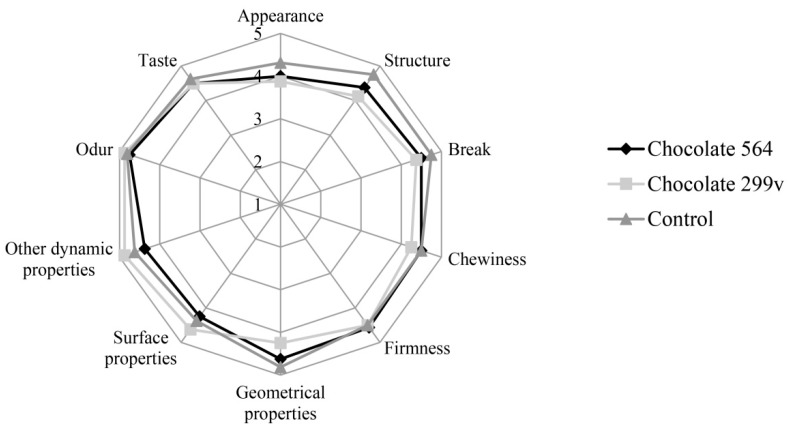
Sensory evaluation of dark chocolates with probiotic bacteria after 60 days of storage at 20 °C.

**Figure 4:**
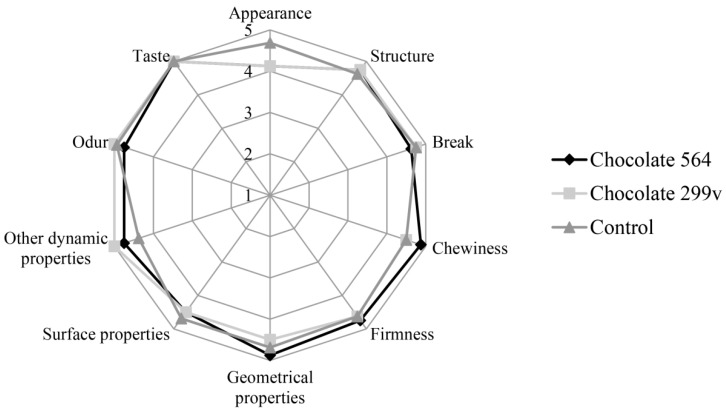
Sensory evaluation of dark chocolates with probiotic bacteria after 180 days of storage at 20 °C.

Considering the fact that both chocolates had the highest probiotic cell number in first two months and that the cells of both probiotic bacteria stayed above 10^6^cfu/g in further 6 months of storage, the sensory evaluation at these two time points was described in this paper. The sensory evaluation of 2 months old samples was shown in Figure 3, while Figure 4 depicts the evaluation of 6 months old chocolate samples.

After 60 days of storage, all three chocolate samples were evaluated with very high marks for odour and taste. Maximum quality of control chocolate was 92.75%, while dark chocolates with spray dried *Lb. plantarum* 564 and 299v gained slightly lower marks (89.66% and 89.06%, respectively). However, probiotic chocolate variants received higher scores for sensory quality after 180 days of storage (95.25% for both experimental varieties), reaching similar value as the control (96.63%). Based on the sensory analysis, it can be concluded that there were not statistically significant differences at the confident level of 0.05 between all three variants during 60 and 180 days of storage at 20 °C. Furthermore, all analysed samples were classified in the excellent sensory quality category.

In this study, sensory evaluation showed that dark chocolate enriched with probiotic bacteria had good marks for appearance and texture. Sandiness, a geometrical property that, if pronounced, could cause texture defects, has not been noticed in both probiotic chocolate varieties during the storage. Also, probiotic dark chocolate had appropriate structure, hardness and good chewiness. Some probiotic bacteria could produce components which may result in the appearance of some foreign odour and taste, such as the so-called vinegar flavour, that may contribute negatively to the taste and aroma of the product [52]. In this experiment, those negative characteristics have not been detected, marking dark chocolates as samples with very good odour and taste during the whole 6 months storage period.

Generally, it can be concluded that both probiotic dark chocolates had excellent sensory quality and that encapsulated probiotic bacteria did not have an effect on aroma and texture of final products in 6 months storage period.

## 4. Conclusions

This study demonstrated that encapsulated spray dried probiotic bacteria (both commercial *Lb. plantarum* 299v and potential probiotic *Lb. planatrum* 564) had very good viability in dark chocolate during storage period of 180 days at 20 °C, while it significantly dropped after further storage of 360 days, reducing the shelf life of Chocolate 564 and 299v to six and nine months, respectively. Nevertheless, encapsulated spray dried probiotic bacteria did not lead to the substantial disruption of texture and aroma, showing an excellent sensory quality of probiotic dark chocolate after 180 days of storage. Also, the chemical composition was not statistically different (*p* > 0.05) between control and probiotic dark chocolate samples. Only minute differences were evident in the volatile profiles between the probiotic and the control chocolate at 180 days but these related mainly to differences in abundance of volatiles rather than actual differences in the type of volatile compounds present.

Hence, it can be concluded that the enrichment of dark chocolate with microencapsulated probiotic bacteria gives a functional product with very good sensory and compositional characteristics. Besides, this research has demonstrated that encapsulation by spray drying could provide adequate protection of probiotic bacteria during production and storage of dark chocolate. The present study provides grounds for further investigation on the possibility of using dark chocolate as a carrier of probiotic bacteria into gastro-intestinal environment, where chocolate may be regarded as an alternative way of supplying probiotics to consumers.

## Figures and Tables

**Table 1:** Sensory evaluation of dark chocolate.

Basic Sensory Properties		Weight Coefficient	Score	Description of the Evaluated Property
APPEARANCE	Form Colour Gloss Surface	2.50	5	Appropriate form; spotless colour; smooth, glossy surface; clear print
			4	Insignificant deviation of form; spotless colour; smooth, glossy surface; less clear print
			3	Deviations of form; lower quality colour; fingerprints on the surface; air bubbles; less clear print
			2	More pronounced form deviations; partially white or grey surface; presence of cracks
			1	Distorted form; grey or white surface; higher damages; bad print
TEXTURE	MEHANICAL PROPERTIES Structure Break Hardness Chewiness	2.50	5	Straight break, homogenous, fragile; homogenous structure; appropriate chewiness
			4	Uneven break; homogenous structure; appropriate hardness; very good chewiness
			3	Uneven break, air bubbles; inappropriate hardness; fat bloom appearance on the break; average chewiness
			2	Uneven break; roughly-granular texture; fat bloom on the break; stickiness; chewiness satisfactory
			1	Crumbling; texture roughly granular; fat bloom; very bad chewiness and very strong stickiness
	SURFACE PROPERTIES Moisture Lubricity	1.0	5	Without surface extracted water/fat
			4	Slight separation of water/fat on the surface
			3	Separation of water/fat on the surface
			2	Clearly expressed separation of water/fat on the surface
			1	Very pronounced separation of water/fat on the surface
	OTHER DYNAMIC PROPERTIES Solubility	0.5	5	Inherent gradual solubility
			4	Slight deviation from the characteristic, gradual dissolution
			3	Deviation from the characteristic, gradual dissolution
			2	Clearly pronounced deviation from the characteristic, gradual dissolution
			1	Very pronounced deviation from the characteristic, gradual dissolution—very slow or very fast solubility
AROMA	THE ODOUR	4.00	5	Appropriate; round; aromatic
			4	Appropriate; less round; aromatic
			3	Appropriate; less round; poorly aromatic
			2	Not appropriate; sour; staled
			1	Foreign odour; sour; staled; mouldy
	THE TASTE	7.00	5	Appropriate; round; aromatic
			4	Appropriate; less round; aromatic
			3	Poorly round; poorly aromatic
			2	Slightly sour; not round
			1	Foreign taste; sour; bitter

**Table 2:** Chemical composition of dark chocolates made with and without probiotic bacteria.

Compositional Parameters	Chemical Composition (g/100g) *		
	Control Variant	Chocolate 564	Chocolate 299v
Moisture	0.82 ± 0.05	0.80 ± 0.05	0.81 ± 0.07
Protein	9.88 ± 0.08	9.86 ± 0.04	9.90 ± 0.06
Lipids	39.94 ± 0.07	39.94 ± 0.06	40.02 ± 0.09
Carbohydrate	24.85 ± 0.09	25.01 ± 0.1	24.84 ± 0.1
Ash	2.30 ± 0.04	2.33 ± 0.07	2.41 ± 0.07

* Values presented are the means of three replicate trials ± standard deviation.

**Table 3:** Volatile compounds detected in dark chocolate with and without probiotic bacteria.

Volatile Compounds	CAS No.	Odour Descriptor	Control Variant	Chocolate 299v	Chocolate 564
			Abundance Levels *		
**Aldehydes**					
Isobutyraldehyde	78-84-2	Banana, malty, chocolate-like, cocoa	9.34 × 10^8 a^	3.88 × 10^8 b^	6.87 × 10^8 ab^
3-Methyl-butanal	590-86-3	Malty, powerful, cheese, green, dark chocolate, cocoa	1.05 × 10^9 a^	4.11 × 10^9 b^	4.57 × 10^9 b^
Hexanal	66-25-1	Green, slightly fruity, lemon, herbal, grassy, tallow	1.37 × 10^8 a^	4.28 × 10^8 b^	4.23 × 10^8 b^
Benzaldehyde	100-52-7	Bitter almond, sweet cherry	2.26 × 10^8 a^	3.84 × 10^8 a^	6.25 × 10^8 a^
Nonanal	124-19-6	Green, citrus, fatty, floral	4.02 × 10^8 a^	1.76 × 10^8 a^	1.72 × 10^8 a^
Isodihydro-lavandulal	35158-25-9	Herbal, lavender, woody, green, blueberry, tomato	1.33 × 10^8 a^	4.29 × 10^8 b^	4.51 × 10^8 b^
Ethyl Vanillin	121-32-4	Sweet, creamy, vanillia, caramellic	2.91 × 10^9 a^	2.85 × 10^9 a^	2.76 × 10^9 a^
**Alcohols**					
Ethanol	64-17-5	Dry, dust	1.88 × 10^9 a^	1.91 × 10^9 a^	1.82 × 10^9 a^
2,3-Butanediol	513-85-9	Fruity, creamy, buttery	7.20 × 10^10 a^	5.18 × 10^10 a^	6.23 × 10^10 a^
1,6-Heptadien-4-ol	2883-45-6	Unknown	5.58 × 10^8 a^	2.28 × 10^8 a^	4.61 × 10^8 a^
2-Heptanol	543-43-7	Fresh lemon grass herbal sweet floral fruity green	2.34 × 10^8^ ^a^	0 ^b^	0 ^b^
2-Ethyl-1-hexanol	104-76-7	Animal, Cardboard	2.20 × 10^8 a^	6.35 × 10^7 b^	2.32 × 10^7 b^
Phenylethyl Alcohol	60-12-8	Unclean, rose, violet-like, honey, floral, spicy	4.35 × 10^8 a^	3.84 × 10^8 a^	4.25 × 10^8 a^
**Ketones**					
Acetone	67-64-1	Earthy, wood pulp, hay	1.63 × 10^9 ab^	1.42 × 10^9 b^	1.89 × 10^9 a^
Butanone	78-93-3	Buttery, sour milk, etheric	1.10 × 10^9 a^	4.09 × 10^8 b^	5.35 × 10^8 b^
Acetoin	513-86-0	Buttery, sour milk, caramel	3.13 × 10^9 a^	1.33 × 10^9 a^	2.74 × 10^9 a^
2-Heptanone	110-43-0	Blue cheese, spicy, Roquefort	7.51 × 10^8 a^	3.90 × 10^8 a^	1.58 × 10^8 a^
Acetophenone	98-86-2	Almond, musty, glue, orange blossom, sweet	1.18 × 10^8 a^	1.68 × 10^8 b^	1.88 × 10^8 b^
2-Nonanone	821-55-6	Malty, fruity, hot milk, smoked cheese	8.12 × 10^8 a^	2.72 × 10^8 b^	3.93 × 10^8 b^
**Pyrazines**					
2-Ethyl-6-methylpyrazine	13925-03-6	Roasted potato	7.93 × 10^7 a^	1.47 × 10^8 b^	1.55 × 10^8 b^
Trimethylpyrazine	14667-55-1	Chocolate, earthy	2.39 × 10^9 a^	3.09 × 10^9 b^	3.17 × 10^9 b^
3,6-Cocoa pyrazine	13360-65-1	Patato, cocoa, roasted nutty	2.52 × 10^8 a^	2.68 × 10^8 a^	2.67 × 10^8 a^
Tetramethyl-pyrazine	1124-11-4	Musty, nutty, chocolate, coffee, cocoa, lard, burnt	6.00 × 10^9 a^	6.30 × 10^9 b^	6.51 × 10^9 b^
2-Methyl-3,5-diethylpyrazine	18138-05-1	Nutty, meaty, vegetable	2.10 × 10^8 a^	1.52 × 10^8 b^	1.55 × 10^8 b^
**Lactones**					
g-Butyrolactone	96-48-0	Creamy, oily, fatty nuances	1.64 × 10^9 a^	6.26 × 10^8 a^	1.35 × 10^9 a^
Pantolactone compound	599-04-2	Unknown	1.63 × 10^8 a^	1.06 × 10^8 a^	7.88 × 10^7 a^
d-Octenolactone	16400-69-4	Sweet, coconut-like	1.47 × 10^8 a^	7.98 × 10^7 b^	7.46 × 10^7 b^
**Acids**					
Acetic acid	64-19-7	Vinegar, peppers, green, fruity floral, sour	8.07 × 10^9 a^	1.12 × 10^10 a^	7.21 × 10^9 a^
Isovaleric acid	503-74-2	Cheesy, sweaty, old socks, rancid, faecal, rotten fruit, goaty	1.16 × 10^10 a^	1.10 × 10^10 a^	1.08 × 10^10 a^
2-Methyl-butanoic acid	116-53-0	Fruity, waxy, sweaty-fatty acid	3.01 × 10^9 a^	2.55 × 10^9 a^	2.59 × 10^9 a^
**Esters**					
Isoamyl acetate	123-92-2	Banana, sweet, pear, apple peel	0 ^a^	4.12 × 10^8 b^	4.32 × 10^8 b^
Phenethyl acetate	103-45-7	Floral, rose-like	2.45 × 10^8 a^	1.77 × 10^8 a^	1.95 × 10^8 a^
**Terpenes**					
β-Pinene	127-91-3	Herbaceous	2.61 × 10^8 a^	1.75 × 10^8 a^	3.52 × 10^8 a^
α-Limonene	138-86-3	Citrus	1.94 × 10^9 a^	4.32 × 10^8 b^	3.79 × 10^8 b^
**Phenols**					
Phenol	108-95-2	Medicinal	1.91 × 10^8 a^	5.83 × 10^7 a^	2.12 × 10^7 a^
**Furans**					
2-Pentylfuran	3777-69-3	Fruity, green, earthy, beany, vegetable, metallic	2.21 × 10^7 a^	1.76 × 10^8 b^	1.83 × 10^8 b^

* Values presented are the means of three replicates; Values within a raw not sharing a common small superscript letter (a, b) differ significantly (*p* < 0.05).
